# Beyond the first bout: Adaptations to repeated injuries across physiological and pathological conditions

**DOI:** 10.14814/phy2.70929

**Published:** 2026-05-26

**Authors:** Cory W. Baumann, Christopher P. Ingalls, Kazunori Nosaka, Jarrod A. Call

**Affiliations:** ^1^ Ohio Musculoskeletal and Neurological Institute Ohio University Athens Ohio USA; ^2^ Institute for Molecular Medicine and Aging Ohio University Athens Ohio USA; ^3^ Department of Biomedical Sciences Heritage College of Osteopathic Medicine, Ohio University Athens Ohio USA; ^4^ Department of Kinesiology and Health Georgia State University Atlanta Georgia USA; ^5^ School of Medical and Health Sciences Edith Cowan University Joondalup Western Australia Australia; ^6^ Department of Physiology and Pharmacology University of Georgia Athens Georgia USA; ^7^ Regenerative Bioscience Center University of Georgia Athens Georgia USA

**Keywords:** aging, eccentric contractions, muscular dystrophy, repeated bout effect, skeletal muscle adaptation

## Abstract

Skeletal muscle exhibits remarkable plasticity following injury, yet most research has focused on responses to a single bout of eccentric contractions. This review addresses adaptations to repeated eccentric contraction–induced injuries across physiological and pathological conditions, with emphasis on insights from preclinical rodent models. In healthy muscle, the repeated bout effect (RBE) reduces strength loss and accelerates recovery after subsequent bouts. However, these adaptations are highly condition dependent. Aging can attenuate the RBE, while dystrophic muscle remains vulnerable to repeated injury despite compensatory remodeling. Other factors, including but not limited to, chronic alcohol intake and malignant hyperthermia can influence these responses, though their effects vary and do not universally abolish adaptation. Collectively, these findings highlight that the trajectory of muscle adaptation depends on its physiological state and underlying pathology. Understanding these condition‐specific mechanisms is essential for developing targeted strategies to optimize recovery, maximize adaptations, and preserve muscle health across diverse populations.

## Graphical abstract

The repeated bout effect (RBE) is an adaptive response to a subsequent injury; muscle physiological or pathological state determines the magnitude and trajectory of strength adaptation.

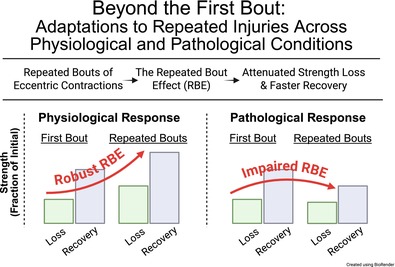

## 1 INTRODUCTION

Skeletal muscle possesses a remarkable ability to withstand and recover from injury. Research using a single bout of eccentric (lengthening) contractions has significantly advanced our understanding of the physiological, cellular, and molecular mechanisms involved in injury susceptibility, repair, and regeneration. However, such an isolated event does not fully represent real‐world conditions, where muscles are often subjected to repeated insults. Indeed, most have focused on acute responses to a single injurious event, overlooking the cumulative and dynamic nature of muscle adaptations, especially outside the context of young, healthy muscle. This review addresses this gap by presenting evidence on skeletal muscle adaptations following repeated bouts of eccentric contraction‐induced injuries. We challenge the notion that weakness and impaired recovery stem from uniform mechanisms across conditions. Instead, outcomes are shaped by the muscle's state at the time of injury and its underlying condition, with distinct mechanisms influencing long‐term function. Recognizing these differences is crucial for advancing our understanding of muscle physiology and pathology.

## 2 HISTORICAL BACKGROUND

Before examining how condition dependent factors influence a muscle's response to eccentric contraction–induced injuries, it is important to understand why this model is widely used in human (and rodent preclinical) research to evaluate functional susceptibility, recovery, and adaptation. Eccentric contractions are well known to cause skeletal muscle injury, typically evidenced by prolonged reductions in muscle function, such as decreased maximal voluntary contraction (MVC) strength (Clarkson et al., 1992). Other markers of injury include delayed onset muscle soreness, increased muscle stiffness and swelling, elevated blood levels of muscle proteins such as creatine kinase and myoglobin, structural disruptions at the myofilament level, and infiltration of inflammatory cells into muscle fibers and the endomysium (Clarkson et al., 1992). Among these indicators, changes in muscle strength are generally considered the most reliable marker of injury when neuromuscular and metabolic fatigue is controlled (Warren et al., 1999), and therefore will be the primary focus of this review.

The human neuromuscular system demonstrates remarkable plasticity; when the same eccentrically biased exercise is repeated, the extent of injury decreases, as reflected by smaller changes in strength and other markers (Clarkson et al., 1992). This adaptive response, where a single bout of eccentric contractions provides protection against subsequent bouts, is known as the repeated bout effect (RBE), a term coined by Nosaka and Clarkson in 1995 (Nosaka & Clarkson, 1995). One of the first comprehensive human studies describing the RBE was conducted by Newham et al. (1987), in which non‐resistance‐trained individuals performed three bouts of voluntary, maximal eccentric contractions of the elbow flexors over 20 min, with each bout spaced 2 weeks apart. Isometric MVC strength decreased by approximately 50% after each bout, but recovery was faster following the second and third bouts compared to the first. Notably, this adaptation in strength recovery was shown to persist for up to 6 weeks (Nosaka et al., 1991).

**FIGURE 1:**
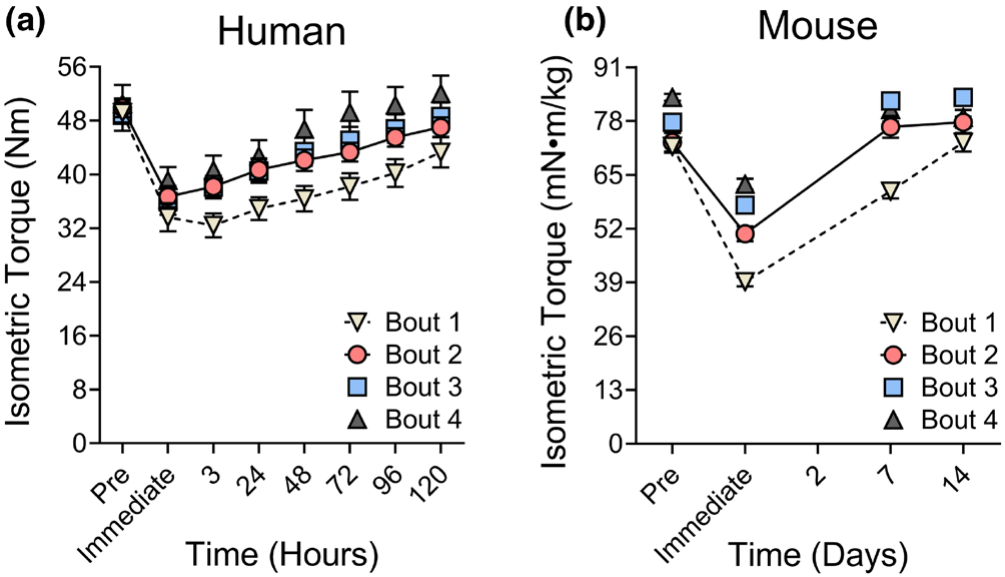
The repeated bout effect (RBE) illustrated by reduced strength loss and faster recovery in human (a) and mouse (b) skeletal muscle. To emphasize that the most substantial adaptation occurs after the first bout, a connecting line (or dashed line) is shown between Bouts 1 and 2. Panel a: Young adult men performed four bouts of 30 voluntary maximal eccentric contractions of the elbow flexors at four‐week intervals; isometric torque was measured for up to 5 days following each bout. Panel b: Young adult male mice underwent four bouts of 150 electrically stimulated maximal eccentric contractions of the dorsiflexors every 2 weeks, with isometric torque assessed for up to 7 days post‐exercise. Data points for Bouts 3 and 4 are displayed individually to illustrate that subsequent adaptations are relatively minor or absent. Panels a and b were re‐created with permission from Chen et al. (2009) and Ganjayi et al. (2024), respectively.

Since the landmark study by Newham et al. in 1987 (Newham et al., 1987), most research on the RBE has limited injury bouts to two. Nearly three decades later, Chen et al. (2009) extended this work by examining four bouts of 30 voluntary, maximal eccentric contractions of the elbow flexors, performed every 4 weeks in non‐resistance‐trained individuals (Figure 1a). Isometric MVC strength decreased significantly after each bout (1st: 34%, 2nd: 27%, 3rd: 24%, and 4th: 22%), with the fourth bout showing smaller strength loss compared to the first and second. A more robust adaptation was observed in recovery; isometric strength returned faster after the second through fourth bouts compared to the first. Interestingly, when resistance‐trained and untrained individuals performed 60 voluntary, maximal eccentric contractions of the elbow flexors, trained subjects exhibited marked protection from initial injury, with MVC strength loss of 25% versus 47% in untrained subjects (Newton et al., 2008). These findings suggest that in human elbow flexors, a single bout of eccentrically biased exercise induces adaptations that enhance recovery of strength, and repeated bouts can further improve protection against strength loss. However, muscles are never fully protected from injury, particularly immediate strength loss, when subsequent bouts are performed at maximal workloads (Figure 1a). The protective adaptations observed after a bout or repeated bouts of eccentric contractions in humans suggest a multifactorial process involving neural, mechanical, and cellular changes (Hyldahl et al., 2017). Importantly, these adaptations not only influence the magnitude of immediate strength loss and the time course of recovery, but may also shape how skeletal muscle responds to subsequent, longer‐term mechanical loading.

Although the RBE is typically viewed as an early, transient adaptation occurring at the onset of training, studying the RBE may have broader relevance for progressive resistance training. Rather than representing an endpoint, the RBE can be conceptualized as a priming stimulus that prepares skeletal muscle for subsequent adaptation. The protective and adaptive responses observed after a single bout may “set the stage” for more robust remodeling by reducing excessive injury, improving tolerance to mechanical load, and enabling greater training volumes or intensities over time. In this context, the RBE may facilitate not only short‐term resilience, but also the accumulation of longer‐term adaptations, including muscle hypertrophy. Thus, a deeper understanding of the RBE lays the groundwork for exploring how physiological and pathological conditions impact muscle adaptations to both acute and chronic repeated injuries, and why functional responses to repeated bouts of eccentric contractions are inherently condition dependent.

## 3 CONDITION DEPENDENT EXAMPLES

The following sections examine both physiological and pathological conditions in which adaptation to repeated, electrically stimulated maximal eccentric contractions have been studied in vivo using preclinical approaches in rodents, primarily mice. We prioritize studies measuring strength loss and recovery across at least two bouts, while noting single‐bout responses only for context and emphasizing repeated injuries, functional adaptation, and brief mechanisms focused on changes beyond the first bout. That said, the transition from human clinical observation to preclinical rodent models requires a high degree of methodological standardization to ensure that findings are both reproducible and translational. As established in a recent Cores of Reproducibility in Physiology (CORP) report (Call et al., 2025), the in vivo skeletal muscle strength approach provides investigators with the ability to longitudinally assess muscle strength in the same research subject. Unlike in vitro or in situ approaches, which are terminal and provide only a cross‐sectional outcome of muscle function, in vivo assessments allow for the repeated, noninvasive measurement of strength production over days, weeks, or months. This longitudinal capability is critical for studying the RBE preclinically, as it allows each animal to serve as its own control, accounting for inter‐subject variability, a core advantage identified in the CORP framework. Furthermore, the use of standardized electrode placement and torque‐frequency curves, as advocated by the CORP guidelines, ensures that the recorded adaptations in recovery and future bout protection are physiological in nature rather than artifacts of measurement error. By utilizing these reproducible in vivo protocols, researchers can record the entire trajectory of the RBE, from the initial mechanical insult to the eventual plateau of adaptive remodeling, across diverse experimental conditions. It should also be noted that this review primarily summarizes findings from mouse studies, as there is limited or no clinical research examining the RBE in the pathological populations outlined here, particularly those for whom maximal eccentric contractions may be contraindicated.

### 3.1 Young and healthy

**FIGURE 2:**
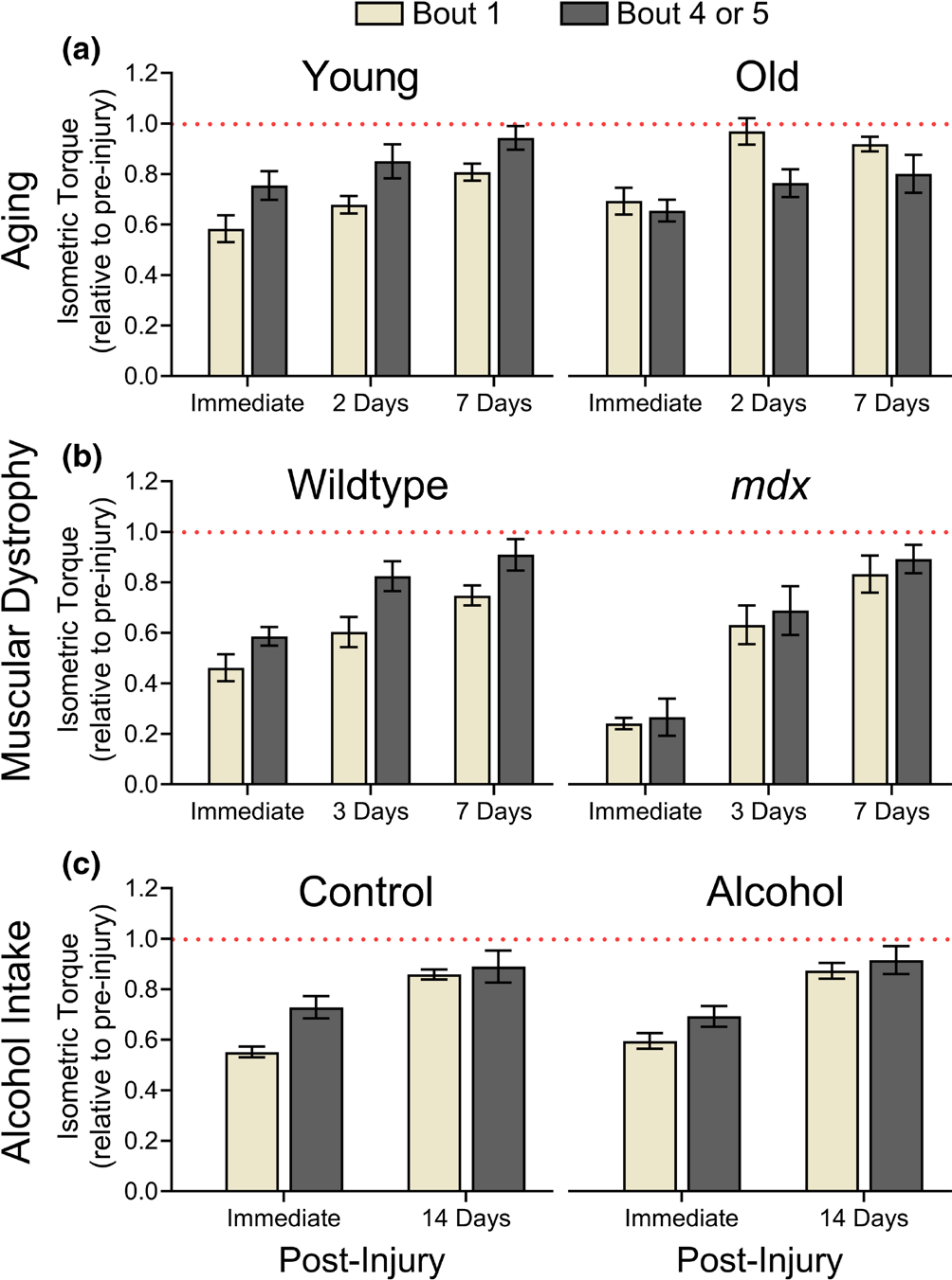
The ability of skeletal muscle to adapt to repeated bouts of injury is often condition‐dependent, as illustrated by mouse models of aging (a), muscular dystrophy (b), and chronic alcohol intake (c). When multiple bouts of electrically stimulated maximal eccentric contractions are performed, these studies suggest that adaptation is diminished in aged muscle and largely absent in dystrophic muscle, whereas chronic alcohol consumption does not impair skeletal muscle adaptation. Panel a: Young and old female mice were subjected to five bouts of 50 eccentric contractions of the dorsiflexors, performed every 7 days. Panel b: Wild‐type (dystrophin‐intact) and *mdx* (dystrophin‐deficient) male mice underwent two bouts of 100 eccentric contractions of the plantarflexors, spaced 14 days apart. Panel c: Control (no alcohol) and alcohol‐consuming female mice (20% ethanol for 24–34 weeks) were subjected to five bouts of 150 eccentric contractions of the dorsiflexors, performed every 14 days. For each condition, maximal isometric torque is expressed relative to pre‐injury torque, which is represented by the dotted red line at 1.0. Data presented were re‐created with permission from Baumann et al. (2023); Panel a, Call et al. (2011); Panel b, and Moser et al. (2023); Panel c.

Similar to human data, most rodent RBE studies report persistent, immediate strength loss following each bout of eccentric contractions, with the most pronounced RBE occurring after the initial bout, both in terms of maximal strength loss and recovery rates (Ganjayi et al., 2024; Ingalls et al., 2004; Lindsay et al., 2021) (Figure 1b and Figure 2; Young, Wildtype, Control). As shown in Figure 1, this attenuation of immediate strength loss is more pronounced in mouse dorsiflexors than in human elbow flexors. However, the protective effect in mouse skeletal muscle is less evident at submaximal compared to maximal isometric contractions (Ingalls et al., 2004). Although not discussed in detail here, it is important to recognize that multiple factors influence the magnitude and duration of the RBE in skeletal muscle, including eccentric contraction intensity and number, strain magnitude and rate, muscle phenotype, and the stimulation frequency used for assessment (Dipasquale et al., 2011; Ingalls et al., 2004; Koh & Brooks, 2001). Accordingly, the studies summarized herein and in Figures 1 and 2 are derived from widely used, well‐accepted protocols and may not necessarily align with every individual study.

Rodent studies allow invasive, muscle‐intrinsic mechanistic investigations of strength loss related to injury and the RBE. Understanding the RBE in young, healthy skeletal muscle requires knowledge of the mechanisms and time course of strength loss after the initial injury bout. Studies, primarily in mouse dorsiflexors subjected to maximal eccentric contractions, indicate that early strength deficits (immediately post‐injury to at least 3 days) are largely due to impaired sarcoplasmic reticulum (SR) Ca^2+^ release (Greising et al., 2025; Warren et al., 2001). This SR dysfunction appears to result from uncoupling between the transverse (T)‐tubule and SR membranes (i.e., excitation–contraction [EC] uncoupling) (Ingalls et al., 1998; Takekura et al., 2001; Yeung et al., 2002), which is associated with loss of triad membrane tethering proteins (Corona et al., 2010). Minor intrinsic dysfunction of the SR Ca^2+^ release channel (RyR1) is also present immediately after injury, potentially due to alterations in RyR1‐binding proteins (Baumann et al., 2014). In addition to impaired SR Ca^2+^ release, early strength deficits partly reflect physical damage to force‐generating or force‐transmitting structures (Greising et al., 2025; Warren et al., 2001). Prolonged weakness, however, appears to stem from focal cellular damage that triggers inflammation‐mediated muscle protein degradation, requiring weeks for recovery and necessitating increased protein synthesis and satellite cell activation (Baumann et al., 2016; Lowe et al., 1995; Rathbone et al., 2003). Thus, early strength loss following eccentric contraction‐induced injury primarily manifest as impaired SR Ca^2+^ handling and EC uncoupling, while prolonged weakness arises from inflammation‐driven structural damage requiring repair, protein turnover (i.e., degradation and synthesis), and regeneration.

Evidence suggests that the RBE involves strengthening of muscle structural and cellular elements, as indicated by reduced muscle fiber degeneration and inflammation during subsequent injury bouts (Deyhle et al., 2020; Lapointe et al., 2002; Corona et al., 2008b). However, because immediate deficits in maximal isometric strength persist, SR Ca^2+^ handling disruptions and EC uncoupling likely remain principal drivers of early strength loss regardless of injury bout (Ingalls et al., 2004; Tabuchi, Poole, & Kano, 2025), rather than persistent physical damage to force‐generating or force‐transmitting structures. Adaptations in EC coupling probably occur to some extent and contribute to the RBE, as attenuated immediate strength deficits across bouts coincide with improved SR Ca^2+^ handling (Tabuchi, Kikuchi, et al., 2025) and increased triad and plasma membrane protein content (Sidky et al., 2022). Faster recovery with the RBE involves two interconnected mechanisms: [1] protection of injured fibers via removal of stress susceptible components by the first bout, and [2] enhanced clearance and resynthesis of damaged proteins during subsequent bouts. In an in vivo mouse model, maximal isometric strength of dorsiflexors recovered faster after five bouts compared to a single bout of eccentric contractions (Ingalls et al., 2004). After the fifth bout, little to no cellular damage or myofibrillar protein loss was observed, suggesting that the muscle was protected or there was a temporal shift in these events (e.g., occurred earlier) when compared to the initial bout (Ingalls et al., 2004). How the initial bout primes muscle for accelerated protein turnover remains unclear, but changes in Ca^2+^ handling may facilitate turnover via time‐dependent calpain activation (Tabuchi, Kikuchi, et al., 2025) and mTORC1‐mediated increases in protein synthesis (Lee et al., 2014). Furthermore, blocking inflammation‐mediated degeneration after the first bout partially prevents adaptations in inflammatory cell infiltration, damage, and faster recovery of strength in subsequent bouts (Lapointe et al., 2002). Overall, rodent studies indicate that the RBE arises from structural reinforcement and improved calcium handling, coupled with enhanced protein turnover that accelerates recovery and limits damage in subsequent bouts.

### 3.2 Increasing age

Skeletal muscle aging is characterized by a progressive decline in strength (Keller & Engelhardt, 2013). Resistance training generally improves muscle function in older adults, resulting in group‐level strength gains; however, these improvements are typically smaller than those seen in younger individuals (Brook et al., 2016; Greig et al., 2011; Lemmer et al., 2000; Macaluso et al., 2000; Raue et al., 2009). Notably, there is also considerable interindividual variability among older adults; nearly one‐third of participants either fail to improve or experience further weakness following training (Clark et al., 2021). These results suggest that the ability to adapt to repeated bouts of exercise may be impaired with age and, in some individuals, could lead to prolonged weakness. Understanding how aging affects susceptibility, recovery, and adaptation after eccentric contraction–induced injuries in skeletal muscle is essential for promoting healthy aging.

Human and rodent studies examining age‐related differences in strength loss and recovery after a single bout of eccentrically biased contractions date to the early 1990s (Brooks & Faulkner, 1990; Dedrick & Clarkson, 1990). However, consensus remains elusive, making it difficult to attribute age‐related differences in long‐term adaptations solely to changes in muscle susceptibility and/or recovery from a single injurious bout (Chapman et al., 2008; McBride et al., 1995; Ploutz‐Snyder et al., 2001; Rader & Faulkner, 2006; Valencia et al., 2021). Consequently, studies investigating repeated bouts and subsequent adaptive processes in both humans and rodents provide essential insights beyond those gained from single bout. To date, only a few studies have assessed the RBE in young and older human subjects, suggesting that older adults may exhibit a diminished RBE or no difference compared to younger individuals (Gorianovas et al., 2013; Lavender & Nosaka, 2006; Skarabot et al., 2019). However, these studies were limited to two bouts, and the observed age‐related differences may have been influenced by the severity of initial injury.

Rodent studies have further advanced our understanding of how age affects the RBE. Although findings vary regarding strength loss and recovery after a single bout, numerous groups report that older muscles adapt less effectively to repeated bouts of maximal eccentric contractions. For example, dorsiflexors of adult (7–9 months) and old (22–24 months) female mice performing six bouts of 180 eccentric contractions at 7‐day intervals both showed some level of protection, but older muscle adapted later and to a lesser degree, with differences emerging after the third bout (Brooks et al., 2001). Similarly, in a subsequent study, dorsiflexor strength of old female mice (27–29 months) initially matched or exceeded young mice (3–5 months) over two bouts of 50 eccentric contractions, but after three additional bouts, exhibited a blunted response, ultimately becoming weaker (Baumann et al., 2023) (Figure 2a). Age‐related deficits in the RBE have also been observed in rat dorsiflexors exposed to 80 eccentric/concentric contraction cycles three times per week for 4.5 weeks (Rader & Baker, 2017). In one study (Cutlip et al., 2006), isometric strength was initially similar between young (3–4 months) and old (31–32 months) male rats, but differences appeared after the fourth bout and widened throughout the study, with young muscle showing a 25% increase in isometric strength and old muscle exhibiting a 34% decrease. Collectively, these findings suggest that age‐related deficits in the RBE reflect a fundamental decline in the muscle's adaptive capacity.

Repeated skeletal muscle injuries amplify age‐related functional differences, offering insight into the mechanisms underlying this effect. Rodent studies indicate that old muscle exposed to repeated, maximal eccentric contractions adapts less effectively than young muscle. From a methodological standpoint, longitudinal in vivo protocols, as outlined in the CORP framework (Call et al., 2025), are essential because aged animals exhibit greater biological variability in baseline strength. Thus, tracking the same subject across multiple bouts allows investigators to distinguish low baseline force capacity from a true failure of the adaptive trajectory, differences that cross‐sectional designs often obscure due to between‐subject variance. Mechanisms for the age‐associated blunting of the RBE are likely multifactorial but have been shown to include (but are not limited to) reduced oxidant buffering capacity, elevated hydrogen peroxide levels, and lower heat shock protein expression (Rader & Baker, 2017) that can subsequently impair muscle quality (Baumann et al., 2023; Cutlip et al., 2006). From a broader perspective, differential gene expression analyses reveal that while young and old muscles share some transcriptional responses to eccentric exercise training, others are age‐dependent (Baumann et al., 2023; Naimo et al., 2019). Studies such as these highlight that aging alters both physiological and molecular responses to repeated bouts of injurious eccentric contractions.

### 3.3 Muscular dystrophy

Rehabilitation strategies and physical activity guidelines for individuals with Duchenne muscular dystrophy (DMD), a progressive muscle‐wasting disorder caused by the absence of dystrophin, emphasize supervised, low‐resistance exercises. These interventions aim to preserve range of motion, minimize contractures, and support functional independence by teaching alternative strategies for task completion (Birnkrant et al., 2018). While preclinical studies using the dystrophin‐deficient *mdx* mouse often report improvements in muscular strength and endurance following exercise (Markert et al., 2011), meta‐analyses of randomized controlled trials in humans suggest that evidence for beneficial muscle remodeling from exercise in patients with DMD remains limited or weak (Hammer et al., 2022; Leone et al., 2024). This discrepancy likely stems from a lack of standardized outcome measures, insufficient studies evaluating optimal exercise dosing (frequency, intensity, and duration), and an incomplete understanding of the mechanisms driving strength loss and adaptation in dystrophic muscle.

Maximal eccentric contractions are particularly detrimental in *mdx* mice, causing significantly greater strength loss in dorsiflexor and plantarflexor muscles compared to wild‐type controls under similar conditions (Baumann et al., 2020; Call et al., 2013) (Figure 2b). Accordingly, such contractions are not recommended in patients with DMD (Birnkrant et al., 2018). Mechanistically, strength loss in *mdx* muscle correlates strongly with a loss of plasmalemmal electrical activity during eccentric contractions (*r* ^2^ ≥ 0.96) (Baumann et al., 2021; Call et al., 2013), alongside an increased frequency of fibers losing their resting membrane potential (Call et al., 2013). Since muscle contraction requires intact membrane potential, plasmalemmal disruption is a key driver of eccentric contraction–induced strength loss in *mdx* muscle. Despite this vulnerability, *mdx* muscle demonstrates a remarkable capacity for recovery (Brooks, 1998; Call et al., 2011), largely attributed to plasmalemmal resealing and restoration of electrical activity (Baumann et al., 2020; Call et al., 2013).

Although a single bout of maximal eccentric contractions can induce transient protective remodeling, *mdx* muscle responds differently due to its inability to restore dystrophin and the dystrophin‐glycoprotein complex essential for membrane stability (Baumann et al., 2021). Repeated bouts of eccentric contractions continue to compromise membrane integrity, leading to substantial strength loss (~60%) even after five exposures (Baumann et al., 2020; Call et al., 2013) (Figure 2b). Nevertheless, *mdx* muscle recovers efficiently from these injuries, with some but not all studies reporting accelerated recovery (Baumann et al., 2020; Call et al., 2011). Intriguingly, despite persistent susceptibility to injury, *mdx* plantarflexor torque was 38% greater after it recovered from four bouts of 100 maximal eccentric contractions (Call et al., 2011). Similar strength gains have been observed in dysferlin‐deficient mice (used to study dysferlinopathies), with a 30% increase in tibialis anterior torque following eccentric exercising training (Begam et al., 2020). These findings suggest that while the RBE is generally absent in dystrophic muscle, it retains a notable capacity to increase baseline strength following repeated injuries.

### 3.4 Malignant hyperthermia

Malignant hyperthermia (MH) is an inherited disorder of skeletal muscle caused by mutations in RYR1. Although the phenotype is usually silent, exposure to certain chemical agents (i.e., halogenated gas anesthetics) lead to excessive SR Ca^2+^ release, triggering hypermetabolism, rhabdomyolysis, and, in severe cases, organ failure and death (Rosenberg et al., 2007). MH has been associated with exertional heat illness and exercise‐induced rhabdomyolysis, implicating thermal and mechanical stress as potential triggers (Davis et al., 2002; Wappler et al., 2001). However, findings from a mouse carrying the MH‐associated Y522S mutation in RYR1 (RYR1^Y522S/wt^) challenge this assumption (Corona et al., 2008b). Following a single bout of 150 eccentric contractions of the dorsiflexor, RYR1^Y522S/wt^ muscle exhibited less injury and recovered more quickly than wild‐type controls. These protective effects persisted across four repeated bouts spaced 7 days apart. It was proposed that the Y522S mutation enhanced the muscle fibers' intrinsic resistance to injury and promoted accelerated recovery, potentially through altered RYR1 Ca^2+^ leak, oxidation or increased protein turnover. Thus, despite initial protection and accelerated recovery from eccentric contraction‐induced injury, RYR1^Y522S/wt^ muscle still demonstrated the capacity to adapt and exhibit the RBE.

### 3.5 Alcohol‐related myopathy

Alcohol is known to suppress skeletal muscle protein synthesis (Steiner & Lang, 2015), raising concerns about impaired recovery from eccentric exercise (Parr et al., 2014); however, most human studies report no significant impact on strength recovery after acute consumption following a single bout (Clarkson & Reichsman, 1990; Hayashi & Tanaka, 2024). These findings may not reflect real‐world conditions, where alcohol is consumed chronically and muscles experience repeated injuries. Because recovery from eccentric contractions depends on protein synthesis (Baumann et al., 2016), chronic alcohol use would theoretically hinder adaptation and lead to weakness. Yet, in a mouse model combining chronic alcohol intake with repeated eccentric contractions, neither short‐ nor long‐term alcohol exposure impaired dorsiflexor strength loss or recovery, even after several bouts of 150 contractions (Moser et al., 2023) (Figure 2c). Female mice displayed baseline myopathy, with approximately 10% lower peak strength, but their ability to adapt was comparable to controls. These results suggest that while chronic alcohol use may cause baseline weakness, repair mechanisms and the RBE remain intact in adult mice, with the stimuli of the eccentric contractions being sufficient to overcome any potential alcohol‐induced deficits in protein synthesis.

## 4 ADDITIONAL FINDINGS ENABLED BY THE RBE

The focus thus far has been on the RBE in physiological and pathological contexts. The RBE has also been used to probe the functional significance of specific proteins in transgenic mice (Corona et al., 2008a; Ganjayi et al., 2024), assessing whether a given protein contributes to the effect, and to evaluate interventions designed to improve or restore this adaptive response. From an applied standpoint, Brown et al. (2023) hypothesized that prolonged, high‐dose RAD140 (Testolone), a selective androgen receptor modulator that targets musculoskeletal androgen receptors and promotes protein synthesis (Miller et al., 2011; Yu et al., 2017), would enhance muscle strength, particularly in aged muscle where the RBE is diminished. To test this, dorsiflexor strength was measured in young (3–5 months) and adult (16–18 months) female mice subjected to repeated bouts of eccentric contractions (Brown et al., 2023). Although adult mice recovered more slowly than young mice, RAD140 did not alter strength after the first bout. Unexpectedly, across subsequent bouts, RAD140 suppressed the RBE in both age groups; the effect was most pronounced in young mice, whose strength after the fourth bout was similar to or lower than the first, whereas young controls exhibited the typical RBE (less strength loss and faster recovery). While the mechanisms are not yet defined, RAD140 administration in female mice has been reported to elevate circulating pro‐inflammatory cytokines (Heinze et al., 2025) and induce significant oxidative damage across multiple tissues (Brown et al., 2023), suggesting that its detrimental effects on muscle may be systemic. Nonetheless, these findings indicate that high‐dose RAD140 can impair muscle adaptation to repeated eccentric‐contraction injuries in female mice, with detrimental effects emerging only after repeated exposure.

## 5 CONSIDERATIONS FOR INTERPRETATION AND TRANSLATION

Interpreting the RBE across experimental models and populations requires careful consideration of methodological and biological context. Differences in contraction mode, motor unit recruitment, neural involvement, and stimulation parameters between human and rodent studies can influence both muscle injury and adaptive responses. In addition, biological variables such as sex and pathological status, as well as the use of maximal eccentric loading in vulnerable muscle, may limit direct translation to aging and clinical populations. The following sections outline these considerations to contextualize RBE findings and inform their application to human physiology.

### 5.1 Human versus rodents study design

Interpretation of the RBE across species must account for fundamental differences in muscle activation between voluntary human exercise and electrically evoked contractions commonly employed in rodent models (Call et al., 2025). During voluntary contractions, motor units are recruited according to the size principle, with force output regulated through orderly recruitment and asynchronous rate coding (Lee et al., 2009; Maffiuletti, 2010; Zero & Rice, 2025). In contrast, electrical field stimulation bypasses central motor control, resulting in synchronous motor unit activation that is independent of physiological recruitment order and biased toward larger‐diameter, fast‐fatigable axons located closest to the electrodes.

Importantly, evidence in humans indicates that RBE is mediated, at least in part, by neural adaptations. Several studies have demonstrated a contralateral RBE, whereby unilateral eccentric exercise confers protection against muscle damage in non‐injured or non‐trained homologous muscle (Chen et al., 2016; Howatson & van Someren, 2007; Starbuck & Eston, 2012; Tseng et al., 2020). These findings strongly support a centrally mediated component of RBE, potentially involving altered motor unit recruitment strategies, improved neural coordination, or changes in central motor drive. Such neural adaptations are inherently absent in anesthetized, electrically stimulated rodent preparations, suggesting that the relative contributions of neural and peripheral mechanisms to RBE may differ between species.

Additional translational limitations arise from the frequent use of supraphysiological stimulation intensities and frequencies in rodent studies. For instance, many studies involving anesthetized rodents employ stimulation frequencies up to or even above 200‐Hz (Baumann et al., 2016; Dolan et al., 2022; Marsh et al., 1998; Zwetsloot et al., 2021). However, during natural, conscious locomotion, motor unit firing rates in mice and rats typically average below 125‐Hz (Gorassini et al., 2000; Hadzipasic et al., 2016). In contrast, even during maximal voluntary eccentric contractions in humans, force production remains constrained by central motor drive and physiological motor unit discharge rates; contractions are therefore maximal but not supramaximal. This distinction is particularly relevant for RBE, as supramaximal, electrically evoked contractions may impose greater and more uniform mechanical stress across muscle fibers than typically occurs during voluntary human exercise. Collectively, differences in contraction mode, motor unit recruitment, activation synchrony, neural involvement, and stimulation parameters are likely to influence the magnitude and nature of muscle damage and the adaptive processes underlying RBE in rodent models. While these models are invaluable for elucidating cellular and molecular mechanisms, careful consideration of these factors is essential when extrapolating preclinical findings to human RBE.

### 5.2 Sex differences

Given the well‐established effects of sex hormones (e.g., estradiol) on inflammation, metabolism, and satellite cell regulation (Enns & Tiidus, 2010; Pellegrino et al., 2022; Tiidus, 2003), sex may be a potential modifier of the RBE. However, recent human studies suggest that the magnitude of RBE, at least functionally, is largely similar between sexes. For example, a study examining maximal eccentric contractions of the dorsiflexors (two bouts of 200 maximal eccentric contractions performed 4 weeks apart) reported comparable RBE‐mediated protection in males and females (Bruce et al., 2021). Similarly, a robust RBE has been observed across the menstrual cycle, with comparable protection against eccentric exercise–induced muscle weakness in the elbow flexors (two bouts of 150 maximal eccentric contractions performed 4 weeks apart), regardless of presumed high or low estrogen levels (Rilling & Power, 2025). Rodent data examining sex differences in RBE are comparatively limited but generally align with these human findings. Although sex was not the primary focus of the study, Ganjayi et al. (2024) reported that the magnitude of protection conferred by repeated eccentric bouts was similar between male and female rodents when strength adaptations were assessed. Specifically, changes in tetanic isometric torque following five bouts of eccentric contractions (separated by 14‐day intervals), compared with a single eccentric bout, were similar between sexes.

Evidence from both human and rodent studies indicates that the protective adaptations underlying the repeated bout effect are largely conserved across sexes. Despite known sex hormone–dependent differences in muscle biology, the magnitude of RBE‐mediated protection and strength recovery appears comparable between males and females when eccentric loading is appropriately controlled. These findings suggest that sex is unlikely to be a primary determinant of RBE magnitude, while underscoring the importance of including both sexes to capture potential context‐ or condition‐specific differences.

### 5.3 Eccentric loading in vulnerable muscle

Maximal eccentric contractions are predominantly used in preclinical animal studies, where they are intentionally designed to induce a large, reproducible acute decrement in muscle function. Such exercise‐induced injury models provide a strong and controllable physiological stimulus, allowing investigators to quantify force loss and interrogate membrane and EC coupling disruptions, inflammation, and regenerative responses, including condition‐dependent effects such as those related to aging. In most rodent studies, eccentric protocols are actually supramaximal, externally controlled, and independent of motivation specifically to ensure reproducibility and precision in measuring injury and recovery. These models are invaluable for uncovering cellular and molecular mechanisms of skeletal muscle plasticity that are difficult to isolate in clinical settings. However, because maximal eccentric contractions generate very high muscle forces and substantial disruption to force‐generating and force‐bearing structures, they differ fundamentally from eccentric training paradigms, which are typically designed to accumulate adaptation over repeated bouts while limiting excessive residual dysfunction. As such, the severity of maximal eccentric injury models may limit their translational applicability, particularly for vulnerable clinical populations in whom preserving functional recovery between bouts is essential.

Submaximal eccentric contractions are intended to stimulate mechanotransduction and adaptive remodeling while limiting tissue damage, an approach supported by both clinical and preclinical studies. Acutely, Chen et al. (2007) showed that when young men performed eccentric elbow flexor contractions at 60% and 80%, but not 40%, of MVC 2–3 weeks before a bout at 100% MVC, clear indicators of the RBE were observed, including reduced strength loss and/or faster recovery. In older men, it was later demonstrated that eccentric contractions at only 10% MVC performed 7 days before maximal eccentric contractions were sufficient to attenuate immediate strength loss and enhance recovery (Chen et al., 2013).

Chronically, submaximal eccentric training can increase baseline muscle strength. In young women, 8 weeks of eccentric knee extensor training performed once weekly at ~80% MVC produced greater strength gains than matched concentric training (19% vs. 11%) (Paschalis et al., 2011). Similarly, eccentric resistance training performed once or twice weekly for 12 weeks at 50% of maximal eccentric strength increased lower‐limb MVC strength by 17%–36% in older adults (Baxter et al., 2024). Supporting these findings, aged rats undergoing 4 weeks of submaximal eccentric resistance training showed increases in serial sarcomere number, fascicle length, and muscle strength (Hinks, Vlemmix, & Power, 2024), whereas maximal eccentric loading led to maladaptive remodeling and weakness (Hinks, Patterson, et al., 2024).

These findings support the use of submaximal eccentric contractions as a safe and effective strategy for promoting muscle adaptation and resilience, particularly in populations for whom high‐force eccentric loading may pose increased risk. However, the research discussed here has been largely limited to aging populations, underscoring the need for additional studies to evaluate the efficacy and safety of submaximal eccentric training in other high‐risk populations.

## 6 CONCLUSIONS

The RBE highlights skeletal muscle's remarkable ability to adapt following eccentric contraction‐induced injuries. However, this adaptive response is not consistent across physiological and pathological conditions. In this review, we emphasize that the magnitude and mechanisms of adaptation are highly condition‐dependent, influenced by factors such as age, disease state, and environment. While healthy muscle demonstrates protection and accelerated recovery after a single bout of maximal eccentric contractions, aging and disorders like muscular dystrophy can impair or even suppress these adaptations. Moreover, it is important to acknowledge that in certain conditions, either detrimental or beneficial effects may emerge only after numerous bouts are performed. This suggests that although the RBE may be evident after a single bout, the muscle's ability to respond to subsequent injuries does not always follow the trajectory predicted by the initial adaptation. These findings challenge the notion of a universal pathway for eccentric contraction‐induced strength loss, recovery, and adaptation, underscoring the importance of tailoring (or even considering) therapeutics and specific training strategies. Ultimately, understanding the nuanced responses to repeated injuries will be essential for optimizing interventions aimed at preserving or enhancing muscle health across diverse populations.

## AUTHOR CONTRIBUTIONS

**Cory W. Baumann:** Conceptualization; supervision. **Christopher P. Ingalls:** Conceptualization. **Kazunori Nosaka:** Conceptualization. **Jarrod A. Call:** Conceptualization.

## FUNDING INFORMATION

Funding through the National Institutes of Health (R03‐AG081950 and R01‐AG097530 to CWB and R01‐AR078903 to JAC) and a Glenn Foundation for Medical Research and American Federation of Aging Research Grant (to CWB). CWB acknowledges the support of the Osteopathic Heritage Foundation through funding for the Ralph S. Licklider, D.O., Endowed Faculty Fellowship in the Heritage College of Osteopathic Medicine.

## CONFLICT OF INTEREST STATEMENT

The authors declare that they have no conflict of interest.

## ETHICS STATEMENT

The authors declare that ethical approval and informed consent were not required.
